# Novel insights on new particle formation derived from a pan-european observing system

**DOI:** 10.1038/s41598-017-17343-9

**Published:** 2018-01-24

**Authors:** M. Dall’Osto, D. C. S. Beddows, A. Asmi, L. Poulain, L. Hao, E. Freney, J. D. Allan, M. Canagaratna, M. Crippa, F. Bianchi, G. de Leeuw, A. Eriksson, E. Swietlicki, H. C. Hansson, J. S. Henzing, C. Granier, K. Zemankova, P. Laj, T. Onasch, A. Prevot, J. P. Putaud, K. Sellegri, M. Vidal, A. Virtanen, R. Simo, D. Worsnop, C. O’Dowd, M. Kulmala, Roy M. Harrison

**Affiliations:** 1 Institute of Marine Science, Consejo Superior de Investigaciones Científicas (CSIC), Barcelona, Spain; 2 National Centre for Atmospheric Science Division of Environmental Health & Risk Management School of Geography, Earth & Environmental Sciences, University of Birmingham, Edgbaston, Birmingham B15 2TT United Kingdom; 3 School of Physics, Centre for Climate & Air Pollution Studies, National University of Ireland Galway, University Road Galway, Galway, Ireland; 4 Aerodyne Research, Inc., Billerica, MA USA; 5 Department of Physics, University of Helsinki, P.O. Box 64, 00014 Helsinki, Finland; 6 Leibniz Institute for Tropospheric Research, Permoserstr. 15, 04318 Leipzig, Germany; 7 University of Eastern Finland, Department of Applied Physics, P.O.Box 1627, FIN-70211 Kuopio, Finland; 8 Laboratoire de Météorologie Physique, CNRS-Université Blaise Pascal, UMR6016, 63117 Clermont, Ferrand France; 9 School of Earth, Atmospheric and Environmental Sciences, The University of Manchester, Manchester, UK; 10 Laboratory of Atmospheric Chemistry, Paul Scherrer Institute, 5232 PSI Villigen, Switzerland; 11 Finnish Meteorological Institute, Climate Change Unit, P.O. Box 503, 00101 Helsinki, Finland; 12 Netherlands Organisation for Applied Scientific Research TNO, Princetonlaan 6, 3508 TA Utrecht, The Netherlands; 13 Division of Ergonomics and Aerosol Technology, Lund University, Box 118, SE-22100 Lund, Sweden; 14 Division of Nuclear Physics, Lund University, Box 118, SE-22100 Lund, Sweden; 15 Department of Environmental Science and Analytical Chemistry, Stockholm University, 10691 Stockholm, Sweden; 16 Laboratoire d’Aérologie, Toulouse, France; 17 NOAA Earth System Laboratory and CIRES, University of Colorado, Boulder, USA; 18 Charles University, Faculty of Mathematics and Physics, Dept. of Atmospheric Physcis, Prague, Czechia; 19 Univ. Grenoble-Alpes, CNRS, IRD, INPG, Institut des Géosciences de l’Environnement, Grenoble, France; 20 Univ. Grenoble-Alpes, CNRS, IRD, Observatoire des Sciences de l’Univers, Grenoble, France; 21 European Commission, Joint Research Centre, Institute for Environment and Sustainability, 21027 (VA), Italy; 22 Department of Evolutionary Biology, Ecology and Environmental Sciences, Universitat de Barcelona, Av. Diagonal 643, 08028 Barcelona Catalonia, Spain; 23 Department of Environmental Sciences / Center of Excellence in Environmental Studies, King Abdulaziz University, PO Box 80203 21589, Jeddah, Saudi Arabia; 24 Present Address: European Commission, Joint Research Centre (JRC), Directorate for Energy, Transport and Climate, Air and Climate Unit, Via E. Fermi 2749, I-21027 Ispra, (VA) Italy

## Abstract

The formation of new atmospheric particles involves an initial step forming stable clusters less than a nanometre in size (<~1 nm), followed by growth into quasi-stable aerosol particles a few nanometres (~1–10 nm) and larger (>~10 nm). Although at times, the same species can be responsible for both processes, it is thought that more generally each step comprises differing chemical contributors. Here, we present a novel analysis of measurements from a unique multi-station ground-based observing system which reveals new insights into continental-scale patterns associated with new particle formation. Statistical cluster analysis of this unique 2-year multi-station dataset comprising size distribution and chemical composition reveals that across Europe, there are different major seasonal trends depending on geographical location, concomitant with diversity in nucleating species while it seems that the growth phase is dominated by organic aerosol formation. The diversity and seasonality of these events requires an advanced observing system to elucidate the key processes and species driving particle formation, along with detecting continental scale changes in aerosol formation into the future.

## Introduction

Atmospheric aerosol formed by nucleation is hypothesized to provide an important source of global cloud condensation nuclei (CCN)^1,2^. Such processes have a major influence on the microphysical properties of clouds and the radiative balance of the global climate system^3^. However - despite its importance - atmospheric nucleation is still poorly understood; it is not clearly known whether it is dominated by a single nucleation pathway, or whether multiple different mechanisms are competing with each other.

There is strong evidence that atmospheric new particle formation (NPF) involves clusters with at least one molecule of sulphuric acid^4,5^; but nucleation theories have failed to predict correctly either the observed nucleation rates or their functional dependence on sulphuric acid concentrations. Recently, the complexity and variability of the atmosphere has hindered elucidation of the fundamental mechanism of NPF from gaseous precursors in experiments performed with the CLOUD (Cosmics Leaving Outdoor Droplets) chamber at CERN. The CLOUD chamber enables precise control of experimental parameters and provides the exceptionally clean experimental conditions that are essential when performing experiments with extremely low concentrations of participating vapours. Overall, molecular analysis of the mechanisms of the initial stage (~1–3 nm) reveals that a number of primary vapours may be responsible. Atmospherically relevant ammonia concentrations increase the nucleation rate of sulphuric acid particles by more than a factor of 100 to 1,000^6^. Ions are also expected to enhance nucleation; nevertheless - even with the large enhancements in rate caused by ammonia and ions - the remarkable study of Kirby *et al*. (2011)^6^ concluded that atmospheric concentrations of ammonia and sulphuric acid are insufficient to account for observed boundary layer nucleation. Better agreement with predicted nucleation rates is achieved when including efficient stabilization of the acids by bases such as amines^7^. Indeed, amines strongly enhance nucleation rates already in the low pptv range^8^. The involvement of oxidized organic molecules in the process, alongside sulphuric acid, was proposed in early studies and has been now well-established^9–11^. Additionally, the existence and formation mechanisms of essentially nonvolatile highly oxidised molecules deriving from biogenic organic vapours in the atmosphere were elucidated, both in laboratory studies and in the ambient atmosphere^12,13^. Lately, evidence for the formation of biogenic aerosol particles from highly oxygenated molecules (HOMs) in the absence of sulphuric acid in a large chamber under atmospheric conditions was presented^14^. On the basis of the combined modelling results and experimental data, such low-volatility organic vapours are the key to particle growth at the initial sizes^15^. There is also strong experimental evidence that pure organic nucleation proceeds alongside sulphuric acid-driven nucleation in the free troposphere^16^. Indeed, simulations and a comparison with atmospheric observations show that nearly all nucleation throughout the present-day atmosphere involves ammonia or biogenic organic compounds, in addition to sulphuric acid^17,18^. The new mechanism for organic particle formation without sulphuric acid provides a way to form particles in the pristine preindustrial atmosphere, when the concentrations of sulphuric acid and ammonia were much lower^19^.

In marine and coastal environments - biogenic iodocarbons emitted from marine algae may control the formation of marine aerosols and cloud condensation nuclei^20,21^. Additional marine biogenic components - namely amines and methanesulfonic acid - may also play a role in the process^22^.

Overall, the clustering of vapours is a major source of new particles of ~1–10 nm in diameter, but these small particles must grow in size to act as CCN and influence clouds.

It is well established that oxidation products of volatile organic compounds (VOCs) are important for particle growth^3,23^. As the particles increase in size (~>10 nm), condensation of organic compounds probably becomes increasingly important, although the exact identities of the organic molecules driving the growth of atmospheric particles are largely unresolved. Our understanding on the role of organics and other chemical species in nanoparticle growth is advancing quickly; but there is a considerable gap between modelling and laboratory studies on the one hand, and direct ambient experimental evidence on both new particle formation occurrence and relative chemical composition on the other. So far, there is very limited experimental evidence upon the chemical processes driving that growth, or the chemical composition of the particles comprising the nucleation mode in the atmosphere^3^.

Coordinated field measurement studies of the atmospheric composition and size distribution of aerosols are essential to bridge the gap between these two extremes. This can help to elucidate the processes responsible for the occurrence and the growth of atmospheric nanoparticles. However, the atmospheric real time detection of the frequency and the chemical composition of NPF events is difficult because it requires the expensive, long term deployment of multiple state-of-the-art instruments to make field measurements in different environments. Here - for the first time - we use novel methodologies applied to the most comprehensive dataset for aerosol size distributions and chemical composition available to date (EUCAARI - European Aerosol Cloud Climate and Air Quality Interactions; EMEP - European Monitoring and Evaluation Programme and EUSAAR - European Supersites for Atmospheric Aerosol Research^24–26^). Specifically - by means of real time, NPF ambient measurements - we aim to throw new light upon the nature of particle nucleation processes across Europe. The main objectives of this work were (a) a categorization and quantification of ambient NPF events by means of aerosol size distributions (mobility diameter ~17–30 nm^27,28^; Methods), (b) elucidating the observed spatial regional NPF variability; and (c) identify the real time chemical composition of maturing/grown nucleation mode particles (vacuum aerodynamic diameter, ~30–60 nm^29,30^, Methods).

## Results and Discussion

### Detecting New Particle Formation Events Across Europe

**Figure 1:**
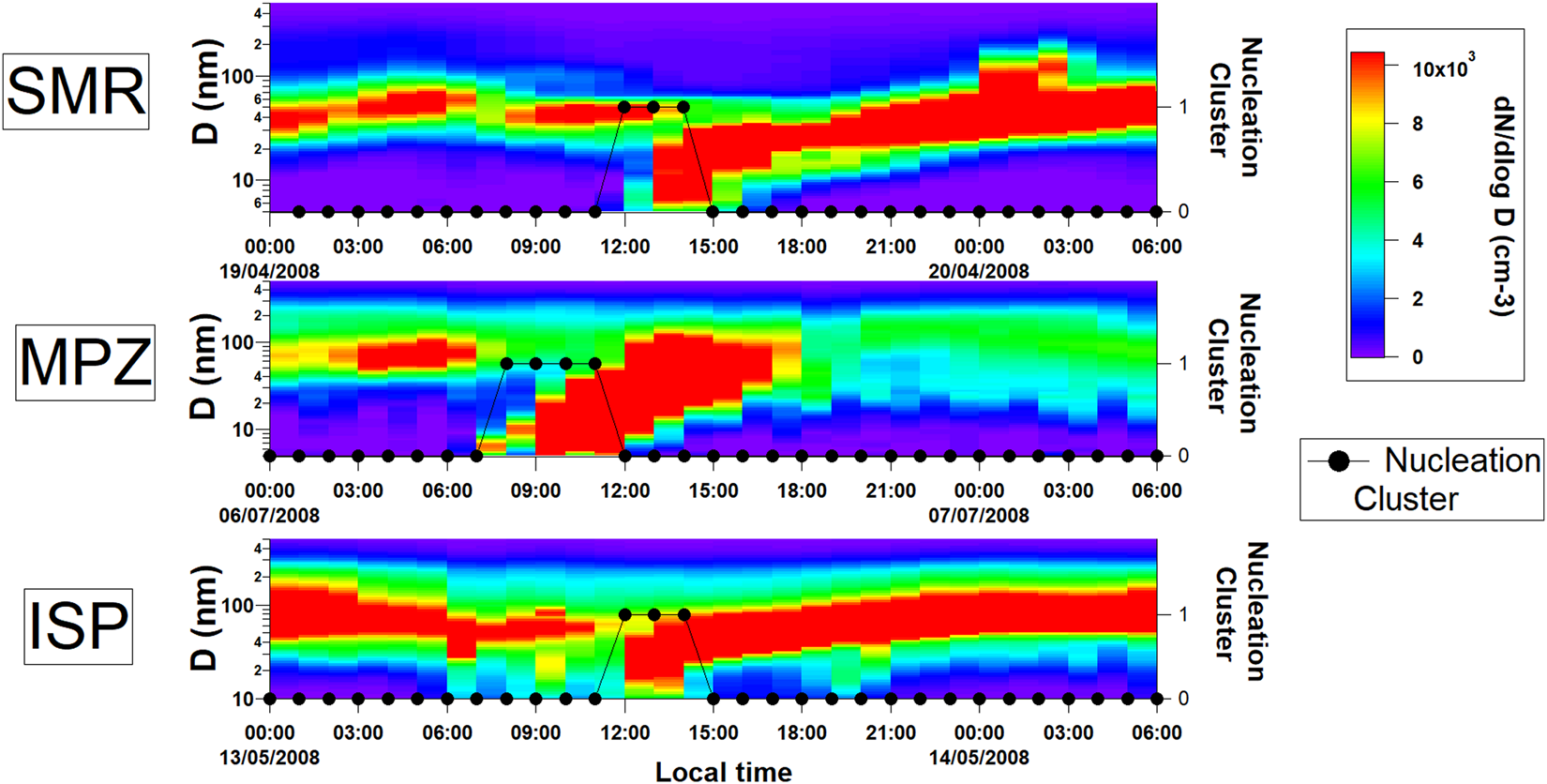
New particle formation events at three sites in Europe. The right hand scale and black dots indicate on a binary scale those hours for which our method attributes the size distribution to the nucleation cluster.

A unique dataset, spanning 24 supersites across Europe was analysed for the frequency of occurrence and seasonal patterns associated with the formation of new particles (Table S1, Methods). The vast dataset, comprising 117,000 hourly size distributions over the years 2008–2009, was exposed to K-means cluster analysis of a maturing nucleation mode size range extending from 17 nm to 30 nm, which in turn, revealed the presence of four clusters describing the entire aerosol population, one of which represents the size distributions associated with a maturing nucleation mode (Methods, Figure S1a-e). An example of how the cluster analysis identifies the occurrence of the nucleation mode is shown in Fig. 1. Previous analyses of the EUCAARI dataset have presented a detailed overview of the sampling sites and seasonally disaggregated size distributions excluding these sizes^27^, or have discussed mainly larger aerosol size modes^28^. Our results are compared with previous continuous atmospheric cluster and particle measurements taken with different types of air ion and cluster mobility spectrometers^31^, and found to be comparable in the overlap particle size region (17–42 nm), as shown in Figure S1f. Our new methodology allows detection in real time of NPF events across the monitoring stations, accounting for 7 ± 4% of the sampled time at each site.

**Figure 2:**
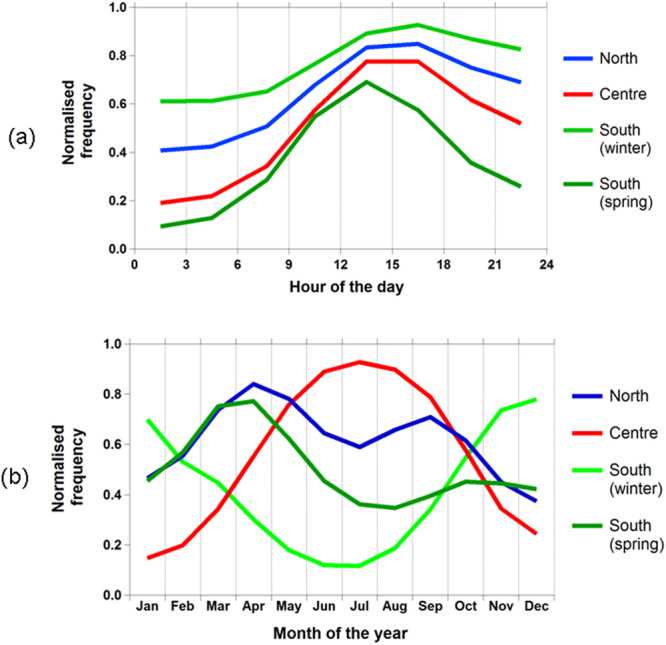
Seasonal prevalence of new particle formation events across Europe in the period 2008–2009 and relative diurnal profile (**a** and **b**, respectively).

**Figure 3:**
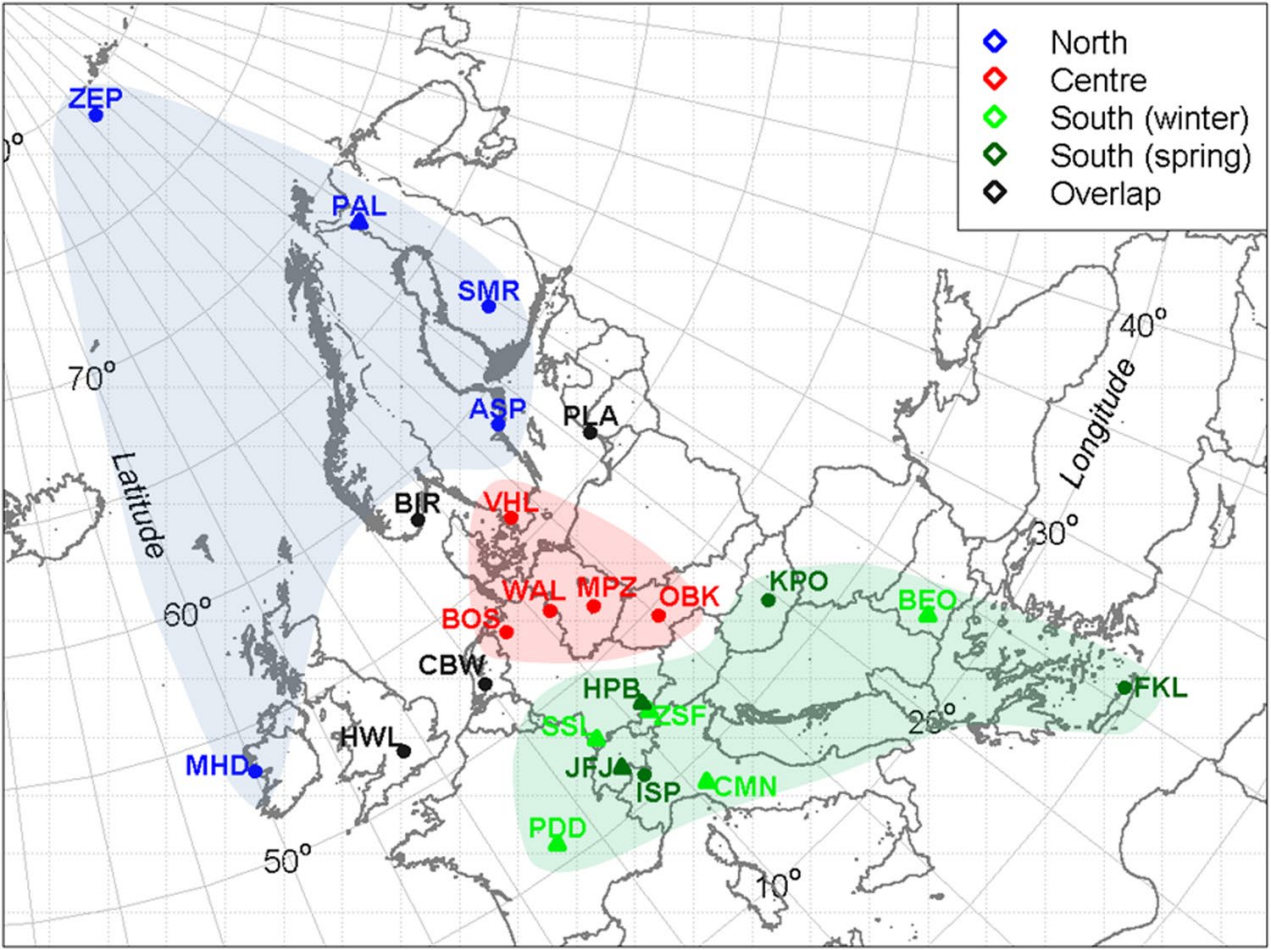
Spatial distribution of the different patterns of new particle formation.

When NPF events for each of the 24 stations were analysed independently, strikingly different seasonal cycles are seen. These are summarised in four main categories, shown in Fig. 2a. The northern group of monitoring stations (ZEP, PAL, SMR, ASP, MHD) show a peak frequency mainly in spring and in autumn. By contrast, central European monitoring stations (VHL, WAL, MPZ, OBK, BIR) mainly show a clear seasonality trend peaking during the summer months. Southern group stations present more complex scenarios, with some having a maximum occurrence in winter (SHC, ZSF, PDD, BEO, CMN) and others in the spring (KPO, FKL, HPB, ISP, JFJ). The average diurnal occurrence of NPF K-mean clusters (Fig. 2b) shows - for all different seasonal categories - a similar profile peaking during daytime^3^, although less sharp for North and South (winter) categories relative to South (spring) and Centre ones. The overall results of our K-means SMPS analysis are summarised and plotted in the map shown in Fig. 3, which is the current best representation of the largest real time aerosol size distribution dataset available today and analysed at a continental level. Overall, we successfully allocated 20 of the 24 studied stations to three broad NPF European regions (North, Centre, South). Four monitoring stations (Black dots; HWL, CBW, BIR, PLA) do not well fit the regional pattern, likely due to an overlap of the geographical regions; or in the case of HWL and CBW experience substantial influence of local anthropogenic sources which may affect their seasonality. For this reason these four stations are categorised as “overlap” and are not discussed in the present work.

### Elucidating the Observed Spatial Regional Variability

Any interpretation of the regionality of the formation of nucleation mode particles must consider both the nucleation process itself and early growth (~1–10 nm), and the subsequent particle growth (~>10 nm); as both processes are an essential pre-requisite to particles being recognised as within the nucleation mode in this study. Thus, factors which need to be considered include the following:

- The condensation sink is a very important factor in influencing the nucleation process. Homogeneous nucleation is unlikely to occur in environments with a high condensation sink as under such circumstances, condensable molecules and clusters are likely to attach to existing surfaces rather than self-nucleating to form new particles.
- Chemical substances which are critical to the nucleation process itself and initial growth (~1–10 nm). The strongest evidence relates to sulphur dioxide as a precursor of sulphuric acid together with basic species (ammonia and amines) but there is also strong evidence for active participation of other species such as oxidised VOC or iodine compounds co-nucleating or acting alone in the nucleation process.
- Chemical substances involved in the growth (~>10 nm) of newly nucleated particles to tens of nanometres in diameter. This requires condensable vapours and the strongest evidence in continental environments is for sulphates and oxidised organic compounds, although other species of low vapour pressure may have the capacity to contribute.

Consequently, the spatial and temporal evolution of the NPF events depends primarily upon two parameters: a high vapour formation rate and a low condensation sink (CS) due to pre-existing particles^32^. In other words, the lifetimes of nucleating particles depend on the competition between their condensational growth and cluster scavenging^33^. The calculated CS is highly variable across the European monitoring stations, although lower in general in north European stations (Figure S2b). The observed meteorological conditions alone (temperature, relative humidity, solar radiation) cannot explain our spatial differences in seasonal behaviour as, although different in amplitude, the same seasonal pattern in meteorology can be seen across Europe (Figure S2a–c). Previous analysis^34^ revealed that while the increased uptake of water by particles does affect the concentration of nucleating vapors and survival of nucleating clusters to some extent, these effects are typically minor in comparison to the reduced OH effect and sulphuric acid limitation.

Nucleation occurred most frequently on sunny days with below-average CS. To test this further, NPF events were explored by correlating nucleation activity and H_2_SO_4_ production - based on an existing concept^35–37^ - Figure S2d shows the relationship between the hourly-average product of [UV intensity * SO_2_ concentration] (a proxy for sulphuric acid production) versus the condensation sink; helping us to view the data in terms of condensation and new particle formation. A total of 3691 hours of specific NPF events from seven sites with available data are plotted. The conclusions that can be drawn from comparing the different NPF events presented in Figure S2d are as follows: for a given level of condensation sink (CS), NPF events in the north of Europe frequently occur at a lower level of SO_2_ concentration than events occurring in central and southern Europe (assuming similar levels of UV, as indicated in Figure S2a). Specifically, for the interval 0.007–0.013 (s^−1^) of CS, average x-values in Figure S2d range by a factor of seven (521, 115, 75 W m^−2^ ppb, for Northern, Central and Southern groups, respectively). The scatter in Figure S2d suggests that the CS, solar radiation and SO_2_ concentrations alone cannot fully explain the variability of the detected ambient NPF events, as pointed out recently^17^.

The variability of vapour phase precursor distributions across the European continent is large. Unfortunately, the only inorganic chemical species continuously monitored in some air quality stations - and thought to be involved in the nucleation process - is SO_2_. In recent decades much lower SO_2_ levels have been recorded following strict implementation of controls upon power generation, manufacturing industry and road transport fuels^38^, although in central and eastern Europe coal-fired power generation plants still play a major role (Figure S3a). The road vehicle fleet is responsible for substantially higher NO_2_ concentrations in Southern Europe than in Western and Northern Europe^39^, as reflected in our data (Figure S3a–c). Ammonia emissions in Europe - 94% originating from agriculture - have fallen since 1990, but by not as much as emissions of other air pollutants^40^.

None of the volatile organic compounds were sampled continuously for the 2008–2009 studied period at the European aerosol monitoring stations, therefore Figure S2d cannot be generated for chemical species other than SO_2_. Nevertheless, we estimated European volatile organic compound emissions calculated by the MEGAN model^41,42^ (Methods) at monthly resolution. Here, we aim to briefly present and discuss the spatial variability of VOC across the three main geographical European regions reported. Biogenic VOC emissions are greater in warmer southern Europe (Figure S3d), although ratios of VOC and Oxidised VOC (O-VOC) are different. When considering the different stations around Europe, the main difference is found between the North and the South regions, whereas the centre shows intermediate values most of the time (S3d–h). Overall, when looking at the ratio of different biogenic VOC between south and north regions (Figure S3e), the South has about two times higher concentrations of VOC relative to the North. Anthopogenic VOC (A-VOC) are also distributed differently (Figure S3g), and also present different seasonal variations^43–45^. Ad-hoc coordinated field studies monitoring both NPF events and organic and inorganic gaseous precursors are needed at continental level in order to address this current large knowledge gap.

### Real Time Chemical Composition of Nucleation Mode Particles

There is limited, but nonetheless useful information upon the chemistry of ultrafine particles during the growth phase of the frequently observed nucleation events across Europe through direct atmospheric ambient measurements. Recently developed research instruments have helped immensely in understanding the processes occurring in the 1–5 nm size range^46–48^. CLOUD laboratory experiments have dramatically advanced knowledge of chemical substances which are critical to the nucleation process itself and initial growth (~1–10 nm), although field observations are limited^16,21^. Further insights into the processes driving particle growth (>~10 nm) can be gained from knowledge of the chemical composition of the nucleation mode particles detected in real time conditions. However, this cannot be discerned from normal air quality observations. In fact, it is difficult to measure it directly with state of the art instrumentation – as usually deployed in research field studies. The best available evidence derives from the Aerodyne Aerosol Mass Spectrometer (AMS)^49^. The time-of-flight (ToF) AMS allows the quantitative measurement of size resolved chemistry of submicrometer non-refractory aerosol with high time resolution and high sensitivity^50,51^. Indeed, the AMS has been successfully applied in a environmental chamber study on new particle formation and growth^20,52^. Additionally - using an AMS - Zhang *et al*. (2004)^29^ were able to show that the composition of the growing particles (33–60 nm vacuum aerodynamic diameter or about 18–33 nm in physical diameter) was predominantly sulphuric acid during the earliest observable stages of formation events in the urban area of Pittsburgh. Also using an AMS in Hyytiala, a forested site in southern Finland, Allan *et al*. (2006)^53^ were also able to characterise by AMS the particles in the <50 nm regime several hours after a nucleation event, demonstrating that the particles were principally organic in composition. However, despite convincing evidence of the presence of organics in growing particles in individual locations, no harmonised analysis of a large region composed of many monitoring stations has been conducted to date.

Therefore, here we use another unique dataset collected over three intensive field measurement campaigns to investigate the aerosol chemical composition over Europe by means of AMS^54^. Such AMS campaigns were carried out also within the framework of EUCAARI/EUSAAR/EMEP during 2008 (May–June and September–October) and 2009 (February–March). Our unique dataset derives from combining the size resolved chemical composition data from the EMEP-EUCAARI-EUSAAR ToF-AMS, temporally overlapped with the presented aerosol size distribution data from SMPS instruments collected over the 24 European monitoring sites. Only 9 of those stations were equipped to collect AMS data and only 5 had size resolved aerosol ToF-AMS data suitable for this study (Table S1). Hourly data were available for both size and composition at KPO (686 hours), MPZ (2,289 hours), PDD (852 hours), and VHL (859 hours). The AMS at SMR did not work during 2008–2009 but a research campaign from May–July 2014 was included (SMR; 2,160 hours). ToF-AMS data were summarised in five logarithmically equally spaced size bins between the range 20 and 500 nm (D_va_, Vacuum aerodynamic diameter), reporting quantitative hourly concentrations of sulphate, nitrate, ammonium and organic matter (Methods). Collection efficiency of the particles at the detector approaches 100% for particles of aerodynamic diameters in the range of 70 to 500 nm, but dramatically decreases for the smaller diameter particles - reaching 0% at about 20–30 nm^49^ (Methods).

Overall, the new combined SMPS-AMS dataset was composed of a matrix of 6,846 hours. Whilst they represent only about 5% of the SMPS dataset, they sum up to about 280 days of combined SMPS-AMS measurements. We applied Positive Matrix Factorization (PMF) to a combination of aerosol size distribution (SMPS) and size resolved chemical composition data (AMS) in order to identify associations between characteristic modes in the size distribution, and the size-resolved chemical composition of particles. This allows us to link directly the chemical composition of particles to size association. Results of the PMF analysis are shown in Figure S4a–c; AMS size bins have been converted from vacuum aerodynamic diameters to mobility diameters^29,30^ using a density of 1.4 g cm^−3^.

Figure S4a–c shows the F-matrix for each of the solutions when we progressively include an additional factor into the model. Starting with a three factor solution, Figure S4aF1-3 shows three number size distribution modes at 25 nm (with a strong organic component), 60 nm (with a strong organic, as well as inorganic sulphate, nitrate and ammonium component) and 150 nm (mainly with an inorganic component composed of ammonium nitrate and sulphate). Adding a fourth factor (Figure S4b), the modal diameter of the factor describing the smallest number size distribution mantains its features. An extra accumulation mode is observed, mainly composed of organic components, but not adding additional information. The additional factor (S4bF3) along with factor S4bF2 likely represent the broad Aitken organic mode represented in the 3 factor solution (S4aF2). The five factor solution also does not additional information, but starts to present similar factors (S4cF2 and S4cF3) as well as not presenting clearly defined particle number size distributions. In summary, the 3 factor solution gives a clear nucleation mode and a clear Aitken mode, as well as an accumulation mode. The nucleation mode shows the strongest association with organic matter in the AMS 30 nm and 55 nm size bins (D_va_).

**Figure 4:**
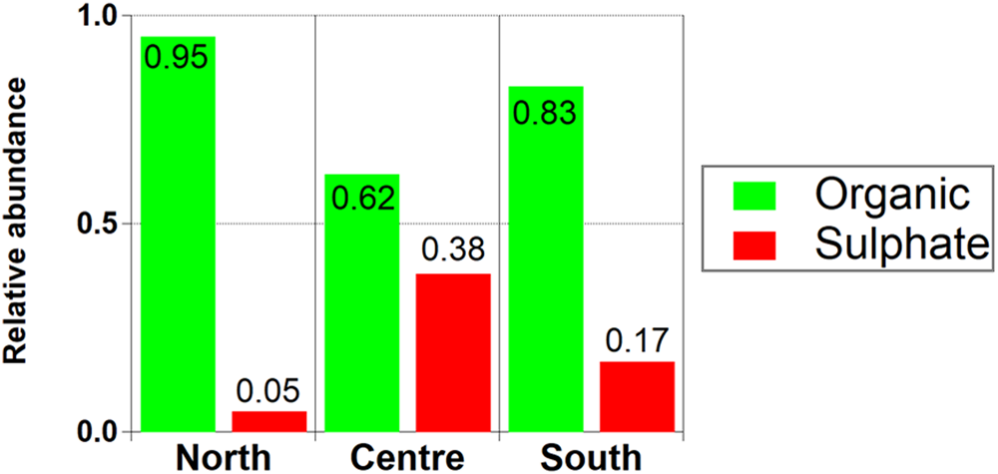
Relative abundance of organic and sulphate in nucleation mode particles (<50 nm) for the three different European regions.

We also looked directly at the ToF-AMS data during new particle formation events. As case studies, we examined 16 events occuring at three stations (SMR (North), PDD (South), MPZ (Centre)), and provide size resolved chemical abundances in the lowest two size bins (vacuum aerodynamic diameter < 70 nm, corresponding to a physical diameter ~50 nm). Figure 4 shows the relative contributions of organics and sulphate to particles in the lowest size range at the three stations. Results point to a major role of organics in particle growth in northern Europe, with the lowest in the Central Europe - where sulphate plays a bigger role, presumably due to higher average concentrations of SO_2_ (Fig. S3a–c). The growth of newly formed particles from sizes of ~1–5 nm up to the sizes of cloud condensation nuclei (~100 nm) in many continental environments requires abundant very low volatility organic vapours^55–57^. Mentel *et al*., (2009)^58^ found that plant-emitted OVOC may play a role in the process of new particle formation consistent with the importance of oxygenated organics in nucleation events over boreal forests^59^. The variation between AMS systems^60^ and the absence of standardized data processing, calibration practices and data treatment in the low diameter AMS size bins creates difficulty in deriving harmonised information at the level of research based field studies^29,30^.

### Inferences

The information presented, and other data presented in the Supporting Information allow some tentative inferences to be drawn regarding the processes likely to determine the seasonal effects upon nucleation/growth events seen clearly in this dataset. These are as below:

- The central group of sites show a peak in nucleation mode particles between June and September (Fig. 2), occurring at midday. These sites show the highest mean concentration of biogenic VOC and similar concentrations of anthropogenic VOC to southern Europe. The condensation sink shows no clear pattern (Figure S2c). It seems likely that at these sites, the nucleation process, favoured by high concentrations of SO_2_ and high insolation favouring its oxidation is the determinant of the seasonal profile.
- The northern group of sites show peak occurrence of nucleation mode particles in April and September and greatest occurrence at midday (Fig. 2). These sites have the lowest sulphur dioxide (Figure S3a) and the lowest concentrations of both anthropogenic and biogenic VOC (Figures S3e and S3g). The condensation sink is low in August and September, favouring nucleation at this time of year, but not in April and May (Figure S2c). As the release of biogenic VOC is low in April following the winter minimum, the explanation for a peak occurrence in Spring is unclear, unless lower temperatures at this time of year are influential. It is notable that several of these sites, most notably MHD, ZEP and ASP are heavily exposed to maritime air masses, and in this context the seasonality of biogenic marine emissions may be important. The contribution of iodine oxides to the nucleation process is known to be important, especially at MHD^20,21^. MSA (methanesulphonic acid) and DMA (dimethylammonium salts) were already identified as a possible connection between marine air masses and particle formation events in North European boreal forests (SMR station^61^).
- The southern group of sites show either a winter or spring maximum in the frequency of nucleation events (Fig. 2a). Those showing a spring maximum (KPO, SIP, HBP, FNK and JFJ) on average show a peak frequency in occurrence at midday in March, while the winter sites (SSL, ZSF, PDD, BEO, CMN) have peaks at midday in December and February (Figure S2a). The southern European sites show intermediate concentrations of sulphur dioxide and high concentrations of nitrogen dioxide (Figure S3a). They show a pronounced summer maximum in biogenic VOC (Figure S3f) and higher levels than other regions. Concentrations of anthropogenic VOC are similar to central Europe. For these sites, the existence of a minimum in the condensation sink at the relevant time of year (Figure S2c) appears to be the probable determinant of frequent nucleation events.

This study shows that new particle formation and growth is a frequent occurrence, but that the processes responsible differ substantially across the European continent. This is the first analysis of the field measurement evidence showing regional differences in nucleation events across a continent, and the first direct evidence that organic compounds dominate the growth of new particles at continental level. The results highlight the importance of a minimum in the condensation sink correlating with a maximum frequency of nucleation at many of the sites. However, this does not explain the higher frequency of nucleation in summer at the central European sites, where high sulphur dioxide and insolation appear to be more important. At many northern European sites, and especially those located close to the coast, it may be marine biogenic precursor concentrations which determine the production of nucleation mode particles. These factors all point to the nucleation, rather than the particle growth process as being the driver of the events when particles reach sizes in the tens of nanometre range from which they can begin to show activity as CCN.

This work highlights some of the benefits which can be gained from coordinated networks of observations. Not only do these yield novel insights into fundamental processes, they also provide the data essential to develop and constrain numerical models of atmospheric new particle formation. The significant costs associated with coordinated European multiplatform atmospheric observational strategies return vastly more information than each of the platforms operating independently. Our work shows that major multiplatform field campaigns and a long term monitoring network are essential to address important research questions.

## Methods

The monitoring sites used to collect data are presented in Table S1 and the reader is referred to ref.^27^ for a full account and description. It is important to note that all these sites are considered as remote or rural regional monitoring sites.

### Measurements of aerosol size distributions

Data were collected using either Differential Mobility Particle Sizer (DMPS) or Scanning Mobility Particle Sizer (SMPS) instruments and then harmonised into a single data set. Further information can also be found elsewhere^28^. Although the instruments within the 24-site network of SMPS/DMPS devices used several different size ranges, all the data collected were harmonised into one large matrix by interpolating the data onto a common size bin scale; 121 size bins spanning 1 to 1000 nm with 40 channels per decade were used. In all, the reduced matrix had 54 size bins (17.8–375.8 nm) and 117 000 hourly particle size spectra (given the capture rates of the instruments across the EUSAAR network in 2008/2009). The data are extracted from the EBAS database (http://ebas.nilu.no), located at NILU (the Norwegian Institute for Air Research), which is a database hosting data for projects and programmes such as the European Monitoring and Evaluation Programme (EMEP), the Global Atmospheric Watch – World Data Centre for Aerosols (GAW-WDCA) and the European Aerosol, Clouds and Trace Gases Research Infrastructure (ACTRIS) network^27,28^.

### Calculation of condensation sink

The condensation sink (CS) describes how rapidly condensable vapour molecules will condense on the existing aerosol. Specifically this quantity describes the loss rate of molecules with diameter d_p_, diffusion coefficient D, and mean free path λ_v_ onto a distribution n(d_p_) (or N_i_ in the discrete case) of existing particles and as such, can be obtained from integrating over the particle size spectrum^62^. Calculations are described elsewhere^63^.

### SMPS K-Means cluster analysis

Although the instruments within the 24-site network of SMPS/DMPS devices used several different size ranges, all the data collected were harmonised into one large matrix by interpolating the data onto a common size bin scale; 121 size bins spanning 1 to 1000 nm with 40 channels per decade were used. More information can be found in ref.^27^. K-means clustering aims to partition n observations into k clusters in which each observation belongs to the cluster with the nearest mean, i.e. given a set of observations (x_1_, x_2_, …, x_n_), where each observation is a d-dimensional real vector, *k*-means clustering aims to partition the n observations into *k* (≤*n*) sets S = {S_1_, S_2_, …, S_k_} so as to minimize the inter-cluster sum of squares (equation 1):

1 $$\mathop{\text{arg}\,\min }\limits_{s}\sum _{i=1}^{k}\sum _{x\in {S}_{i}}{\Vert x-{\mu }_{i}\Vert }^{2}$$

where *μ*_i_ is the mean of points in Si^28,64^. The analysis works given a predefined number of clusters and an optimum needs to be decided upon. In this work, the optimum cluster number was derived using the total residual sum-of-squares (RSS Index) calculated for the clustered normalised data. This was calculated for 2 to 30 clusters and plotted on a log-log graph Figure S1a. The RRS is a measure of the discrepancy between the data and the fitted clusters. A large number of clusters (20–30) will in no doubt produced a small RSS due to very tight fit but will not identify the main clusters in the data – the data will be over fitted. Too few clusters (2–3) will indeed under fit the data and as additional clusters are added the RSS will be reduced. There will however be an optimum number of clusters between these two extreme cases where additional clusters will not produce the same reduction in RSS and this is argued to be the point where the main clusters have been fitted in the data and identified as being the point in the RSS vs cluster number point where a ‘knee’ in the data can be seen. To help identify this point, tangents are drawn on the plot which run along the curve at the low and middle points of the data and where these two meet the ‘knee’ in the data is defined. The first point was located at 5 clusters and the second point was located. To verify this finding, the Calinski and Harabasz (CH) Index was also calculated for the 2–30 clusters and this was observed to be a maximum for 5 clusters when applied to non-normalised data.

### Measurement of Gaseous Pollutants During EUCAARI-ACTRIS

#### Inorganic gases

Standard continuous gas measurements were taken for SO_2_ (UV fluorescence) and NO_2_ (chemiluminescence) as described elsewhere^24–26^.

#### Volatile organic compounds(VOC)

VOC are not measured directly at the 24 EUCAARI stations. Hence, data were obtained by the Model of Emissions of Gases and Aerosols from Nature (MEGANv2.1) together with the Modern-Era Retrospective Analysis for Research and Applications (MERRA) meteorological fields. This dataset is called MEGAN-MACC and allows creation of a global emission dataset of biogenic volatile organic compounds (BVOC) available on a monthly basis for the time period of 1980–2010^42^. Four model grids closest to the EUCAARI stations were chosen, allowing a resolution of 4x (0.5 deg latitude × 0.666 deg longitude).

#### Measurements of particle composition

Mass concentrations and size distributions of submicron nonrefractory sulfate, nitrate, ammonium, and organics were measured with the AMS. Detailed descriptions of the Aerodyne AMS can be found elsewhere^50,51^. The AMS has nearly 100% transmission efficiency for the considered particle size range of 70–500 nm vacuum aerodynamic diameters (Dva), whereas it is lower for the smallest particle sizes (~20–70 nm). No attempt was made to correct the measured size distributions for partial transmission of larger and smaller particles. While this may lead to an underestimation of the growth rate of ultrafine species, it does not affect our ability of identifying the species that are responsible for the growth (Zhang *et al*., 2004; 2005). Although techniques for improving the determination of the NH_4_^+^ size distributions for ultrafine particles exists^29,30^, in this study the two smallest bins (D_va_ 20–38 nm and D_va_ 38–72 nm) were not considered for this chemical species, because low signal-to-noise conditions were experienced for some of the ToF-AMS instruments deployed. Similar to ammonium^29,30^, the removal of gaseous interference from the size distributions was also investigated for organics (m/z 28 CO^+^ and m/z 44 CO_2_^+^). Other m/z generally chosen in particle ToF mode contain negligible amounts of gas signals compared to the particle signals because the aerodynamic lens and skimmers of the AMS reduce the concentration of gas phase species by a factor of 10^7^ relative to aerosol species^65^.

### PMF Analysis of SMPS-AMS Combined Dataset

SMPS data were obtained from previous studies^27,28^. Although the instruments within the 24-site network of SMPS/DMPS devices used several different size ranges, all the data collected were harmonised into one large matrix by interpolating the data onto a common size bin scale; 121 size bins spanning 1 to 1000 nm with 40 channels per decade were used. AMS data were used also from previous studies^54^. Aerosol mass spectrometer (AMS) measurements were carried out during 26 field campaigns at 17 different sites. Only five monitoring stations were overlapping with AMS and SMPS data. Particle time of flight (PToF) AMS data were obtained for nitrate, ammonium, sulphate and organics. Five equally spaced bins were obtained, 20–38 nm, 38–72 nm, 72–137 nm, 137–262 nm, 262–500 nm. More information can be found elsewhere^51^.

PMF analysis was applied to the AMS-SMPS dataset, following the same approach recently described in ref.^66^. Compared to cluster analysis, which groups similar data together, Positive Matrix Factorisation is used to identify the common ‘building blocks’ within the data. PMF solves the general receptor modelling problem using constrained, weighted, least-squares applied to the input data *x* which represent a matrix of concentrations, albeit particle or PM, measured at specific intervals during the study^67^. The general model assumes there are *p* factors F which are interpreted as fixed emission source profiles and impact the receptor site by various amounts - represented by the scores G - during the measurement. PMF determines the profiles of these factors and calculates their contribution G such that the sum of linear combinations G x F of closely matches the measured concentration. Mathematically, the observation *x*_ij_, at the receptor is represented in the matrix equation X = G × F + E whose elements are,

2 $${x}_{ij}=\sum _{h={\rm{1}}}^{p}{g}_{ij}\cdot {f}_{hj}+{e}_{ij}$$

The measurements (AMS or NSD concentrations) are indexed by the integer *j* for the *i*^th^ time step (hour or day). The term *g*_ik_ is the contribution of the *k*^th^ factor to the receptor site on the *i*^th^ hour/day, *f*_kj_ is the fraction of the *k*^th^ factor (AMS or NSD concentrations) that contributes to measurement *j*. Matrix E, comprises of elements *e*_ij_ which are the residual values for the *j*^th^ measurement on the *i*^th^ hour.

In PMF, only *x*_ij_ are known and the goal is to estimate the contributions (*g*_ik_) and the fractions (*f*_ij_). It is assumed that the contributions and number fractions are all non-negative, hence the “constrained” part of the least-squares. Furthermore, PMF uses uncertainties measured for each of the *x*_ij_ size-bin. measurements with high uncertainty are not allowed to influence the estimation of the contribution and fractions as much as those with small uncertainty, thus giving the “weighted” part of the least squares.

Given the above, it is task of PMF to minimise the sum of the squares Q calculated using equation 3.

3 $$Q=\sum _{i={\rm{1}}}^{n}\sum _{j={\rm{1}}}^{m}{(\frac{{e}_{ij}}{{s}_{ij}})}^{{\rm{2}}}$$

where *s*_ij_ is the uncertainty in the *j*^th^ measurement for hour/day *i* and PMF can be operated in a robust mode, meaning that “outliers” are also not allowed to influence the fitting of the contributions and profiles^68,69^.

## Electronic supplementary material

Supplementary Information
