# Supplementary material for: Novel insights on new particle formation derived from a pan-european observing system
Source: Sci Rep. 2018 Jan 24;8:1482. doi: 10.1038/s41598-017-17343-9 (PMC5784154; doi:10.1038/s41598-017-17343-9)
Supplement: Supplementary file 1 — Supplementary Information [file 41598_2017_17343_MOESM1_ESM.pdf]

SUPPLEMENTARY INFORMATION

Novel Insights on New Particle Formation Derived from a Pan-European Observing System

M. Dall'Osto, D.C.S. Beddows, A. Asmi, L. Poulain, L. Hao, E. Freney, J. D. Allan, M. Canagaratna, M. Crippa, F. Bianchi, G. de Leeuw, A. Eriksonn, H. C. Hansson, J. S. Henzing, C. Granier, P. Laj, T. Onasch, A. Prevot, J. P. Putaud, K. Sellegri, E. Swietlicki, M. Vidal,A. Virtanen, K. Zemankova, R. Simo, D. Worsnop, C. O´Dowd, M. Kulmala and Roy. M. Harrison

1. SMPS inspection : Aerosol contribution from urban local sources

The average annual variation and diurnal profiles of the four k-means clusters are shown in Figure S1d. Cluster 3 (nucleation) shows an annual trend peaking in spring (April) and fall (September), and a clear diurnal profile peaking during daylight time (noon). However, cluster 4 was also found to peak during day time.

After inspection of SMPS data, it was found that cluster 4 was also partially composed of aerosol size distributions attributed to late stages of new particle formation growth events. Indeed, the diurnal profile of cluster 4 (Fig S1d) shows a shift in time (peaking at 3pm) relative to cluster 3 (first stage of new particle formation events). Figure S1c also shows a growing mode of the new particle formation mode.

Part of the definition of a remote region is that it is not influenced by traffic or local sources. We wanted to investigate if cluster 3 was affected by ultrafine anthropogenic emissions not related to new particle formation. In order to do so, we selected three monitoring sites representative of monitoring stations situated in polluted continental background areas including: Ispra (ISP, Po Valley, Italy, Southern Europe); K–Puszta (KPO, Hungary, Central Europe) and Kosetice (OBK, Czech Republic, Eastern Europe).

The chart presented in Figure S1e shows the four K-means clusters for two different sets of monitoring stations: "polluted" represents the average of three stations (ISP, KPO, OBK) relative to the average of all sites (ALL). Figure S1e clearly shows that the diurnal profiles differ for polluted, except for Cluster 3 (nucleation). In particular, cluster 2 shows an

enhancement in polluted sites in the 6-9am time interval, suggesting it is the major one affected by anthropogenic emissions. In summary, we conclude that cluster 3 is not affected by local contamination, and can be attributed to new particle formation events.

2. PMF solution of the AMS-SMPS combined dataset

Number Size Distributions (NSDs) data were obtained from Ref. 28. Although the instruments within the 24-site network of SMPS/DMPS devices used several different size ranges, all the data collected were harmonised into one large matrix by interpolating the data onto a common size bin scale; 121 size bins spanning 1 to 1000 nm with 40 channels per decade were used. AMS data were used from Ref 54. Aerosol mass spectrometer (AMS) measurements were carried out during 26 field campaigns at 17 different sites. Only five monitoring stations were overlapping with AMS and NSD data. Particle time of flight (PToF) AMS data were obtained for nitrate, ammonium, sulphate and organics. Five equally spaced bins were obtained, 20-38nm, 38-72nm, 72-137nm, 137-262nm, 262-500nm. More information can be found in Ref. 51.

PMF analysis was applied to the AMS-NSD dataset, following the same approach recently described in Ref. 66. Compared to cluster analysis, which groups similar data together, Positive Matrix Factorisation is used to identify the common 'building blocks' within the data. PMF solves the general receptor modelling problem using constrained, weighted, least-squares applied to the input data *x* which represent a matrix of concentrations, albeit particle or PM, measured at specific intervals during the study[67]. The general model assumes there are *p* factors F which are interpreted as fixed emission source profiles (NSD and AMS spectra) and impact the receptor site by various amounts - represented by

the scores G (time series of the source profiles) - during the measurement. PMF determines the profiles of these factors and calculates their contribution G such that the sum of linear combinations G x F of closely matches the measured concentration. Mathematically, the observation x_{ij}, at the receptor is represented in the matrix equation X = G x F + E whose elements are,

$$x_{ij} = \sum_{h=1}^{p} g_{ij} \cdot f_{hj} + e_{ij} \tag{1}$$

The measurements (AMS or NSD concentrations) are indexed by the integer j for the ith time step (hour or day). The term g_{ik} is the contribution of the kth factor to the receptor site on the ith hour/day, f_{kj} is the fraction of the kth factor (AMS or NSD concentrations) that contributes to measurement j. Matrix E, comprises of elements e_{ij} which are the residual e_{ij} values between the measurement and model for the jth measurement on the ith hour

In PMF, only x_{ij} are known and the goal is to estimate the contributions (g_{ik}) and the fractions (f_{ij}). It is assumed that the contributions and number fractions are all non-negative, hence the "constrained" part of the least-squares. Furthermore, PMF uses uncertainties measured for each of the x_{ij} size-bin. Measurements with high uncertainty are not allowed to influence the estimation of the contribution and fractions as much as those with small uncertainty, thus giving the "weighted" part of the least squares.

Given the above, it is task of PMF to minimise the sum of the squares Q calculated using equation 2.

$$Q = \sum_{i=1}^{n}\sum_{j=1}^{m}\left(\frac{e_{ij}}{s_{ij}}\right)^2 \tag{2}$$

where s_{ij} is the uncertainty in the jth measurement for hour/day i and PMF can be operated in a robust mode, meaning that "outliers" are also not allowed to influence the fitting of the contributions and profiles.

The elements of the matrix S, are derived from the uncertainties entered by the user and these can be entered directly as a matrix using the X_std-dev file. The method chosen for the X_std-dev file values is based on the method used in Ref 68 and Ref. 69. In this, S is calculated using equation 3 (used in PMF2 when selecting EM=-14),

$$s_{ij} = t_{ij} + v_{ij}\max(|x_{ij}||y_{ij}|) \tag{3}$$

Where x_{ij} are the actual data values and y_{ij} are the equivalent data values fitted by PMF. Matrices t_{ij} (sometimes thought of as the the estimated detetion limit – 10-20% of X for the NSD data and 21-233% of X for the AMS data) and v_{ij} (comparable to the relative uncertainty of the measurement - ~6% for the NSD and ~21% for AMS data) are given by

$$t_{ij} = T(x_{ij} + \overline{x_j}) \tag{4}$$

$$v_{ij} = V \tag{5}$$

We chose the empirical values of T and V by trial and error until their calculated Q value was the closest to the theoretical value it could be, i.e. Q/Q_{theory} ~1. For our 3 factor solution, T=V=0.1, impling that the uncertainty is 10% of the maximum of the fitted and

actual values of X and the detection limit of each value is taken as 10% of the sum of that value and the mean of all of the values in the same column. For this case, this formulation gives an uncertainty values between 16 and 27 % for the NSD data 42 and 260 % for the AMS data. Q_{theory} is taken as the difference in the sum of the elements in the input matrix X and output matrices G and F.

In addition to optimising Q/Q_{theory} for the whole data set according to V and T, the Q/Q_{theory} value for both the AMS and NSD data were both adjusted to ~1 by optimising two additional multipliers D_{ams} and D_{nsd}. D_{ams} and D_{nsd} were used to scale the uncertainties of the AMS and NSD matrices respectively with the aim to 'balance' the PMF model. The model is balanced when Q_{NSD} /Q_{t_NSD} ~ 1 and Q_{AMS}/Q_{t_AMS} ~ 1,

So for example, for a our preferred 3 factor solution the closest we could get to a balanced solution was whilst still maintaining Q/Q_{theory} ~ 1 was when using T=0.1; V=0.1; D_{ams}=0.2.1; D_{ams}=0.6.. These values are presented in Table S2 for each solution.

As with Cluster Analysis, optimum settings need to be derived (e.g. Number of Factors) for the model. For a given uncertainty matrix, metrics derived from the residual matrix can be used to give an approximation of the ideal number of factors[68,69]. When deciding on the number of factors a useful constraint is to determine when 'factor-splitting' occurs. This is when a factor which fits a source within the data is forced to divide and can be detected by strong linear relationship between the G scores for the divided factors. Inspection of the scaled residuals and the ratio of Q/Q_{theory} (~1) are also accepted indicators of a good fit where Q_{theory} approximately equal to the number of entries in your data array. However,

more often than not, it is more intuitive to make the final decisions based on how well the model fits the science being explained by the model.

Table S2 shows the settings used for the initial investigations. For each factor number, the t and v values (see equations 3-5) were adjusted until the ratios of Q/Q_{theory}, Q_{NSD} /Q_{t_NSD} and Q_{AMS}/Q_{t_AMS}, were ~1 and after 6 factors evidence of factor splitting became stronger. Hence 3 to 6 factor solutions were considered.

An estimation of how well the model is fitting to the data for each factor setting can be judged by looking at the scaled residual matrix R and in particular calculating IM = (colMeans(R)) the *maximum mean column value of R* and IS = max(apply(R,2,stdev)) the *maximum column standard deviation value of R*. These give a idea of the spread of the residuals after the model has been fitted for each factor number and a smaller number indicates a preferred fit. For the fitted models presented in Table S2, the values of IM decrease by about 23 % when the factor number is increased from 3 to 9, whereas IS increases by 16 %. The ratio of IS to IM, remains around 3.5 from 3 to 5 factors before almost doubling when using 8 factors. On increasing the factor number, the expected trend is for IS and IM to decrease sharply with from 2-5 factors before shallowing off over the higher factors. In comparson the maximum rotation value max(R) increases and using the two a mininimum and maximum factor number can be selected. In this case, because we optimise each model for Q/Q_{theory}, only a maximum factor number can be selected, in this case 5. Hence from the study of IM, IS and max(Rot), factor numbers between 3 and 5 can be chosen as being suitable.

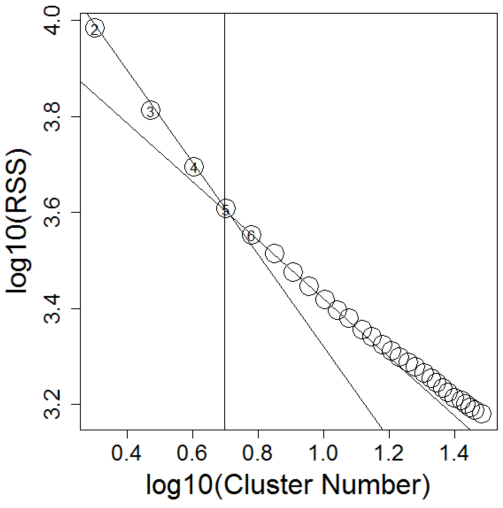

Figure S1a. Plot of Residual Sum of Squares (RRS) against cluster number to identify the main cluster groups within the data. Clusters number 2 to 6 are marked and cluster 5 has been identified as the optimum number of clusters (where the 'knee' in the curve is) using the two tangents marked through the lower 5 and middle 5 to 8 points.

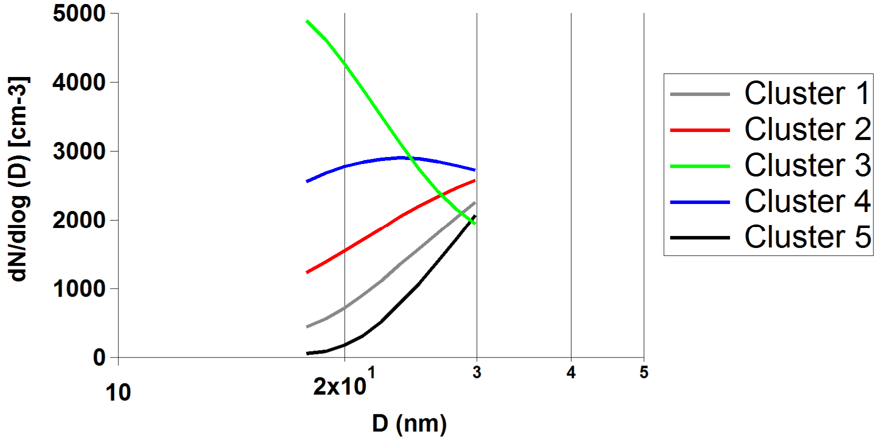

Figure S1b. The aerosol size distributions of the optimum number of clusters. Cluster 1 and 5 were found similar to each other and not relevant for high particle concentrations in the studied size range, so they were merged.

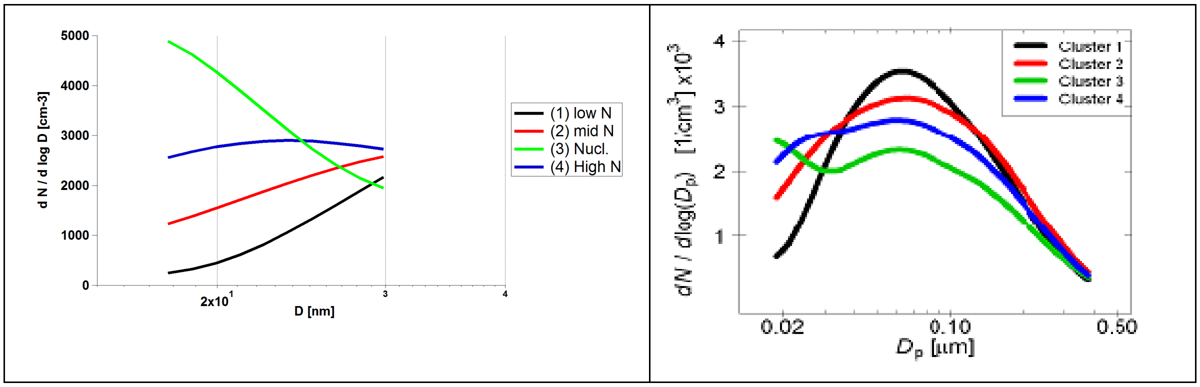

Figure S1c. Aerosol size distributions of the four clusters in the range 17-30 nm resulting from K-means clustering. On the right side the extracted full aerosol size distribution (17-500nm) can be seen. The resultant four factors (1-4) contributed 32±20%, 34±9%, 7±4% and 27±15% respectively of the total size distribution spectra.

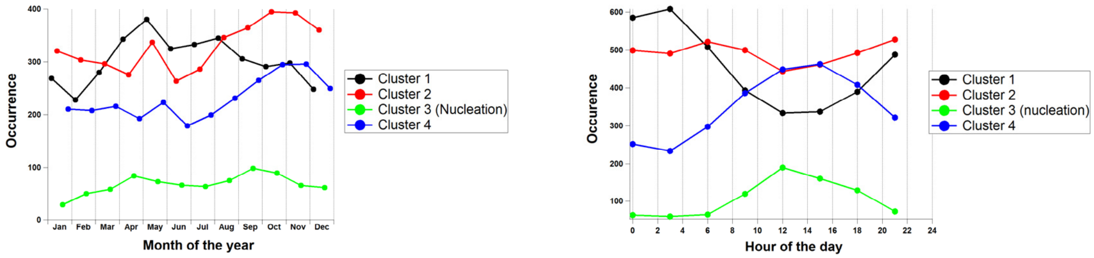

S1d. Annual variation and diurnal profiles of K-means clusters.

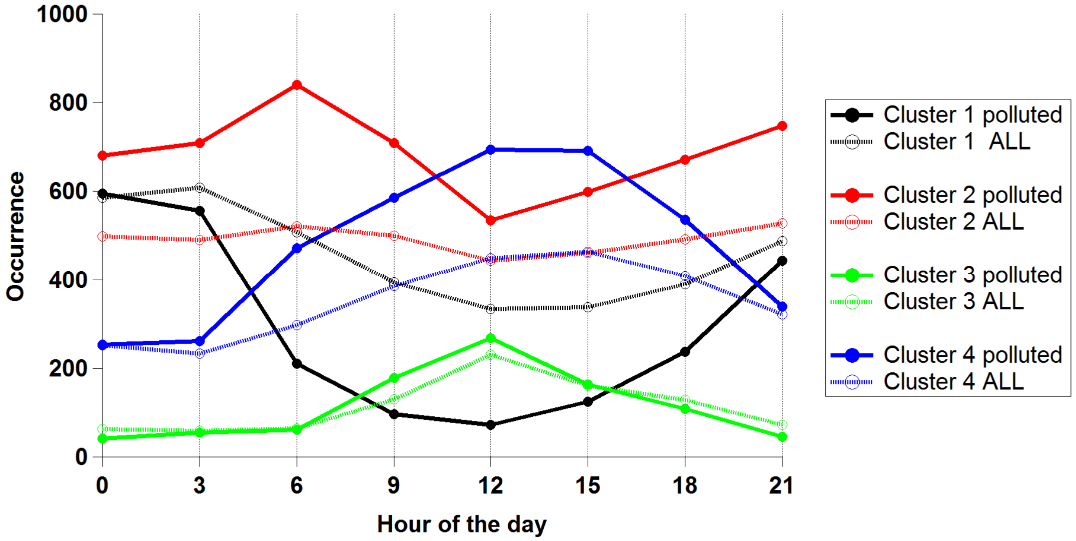

Figure S1e. Monitoring sites influenced by traffic (remote continental stations of ISP, KPO and MPZ) relative to average of all (ALL)**.**

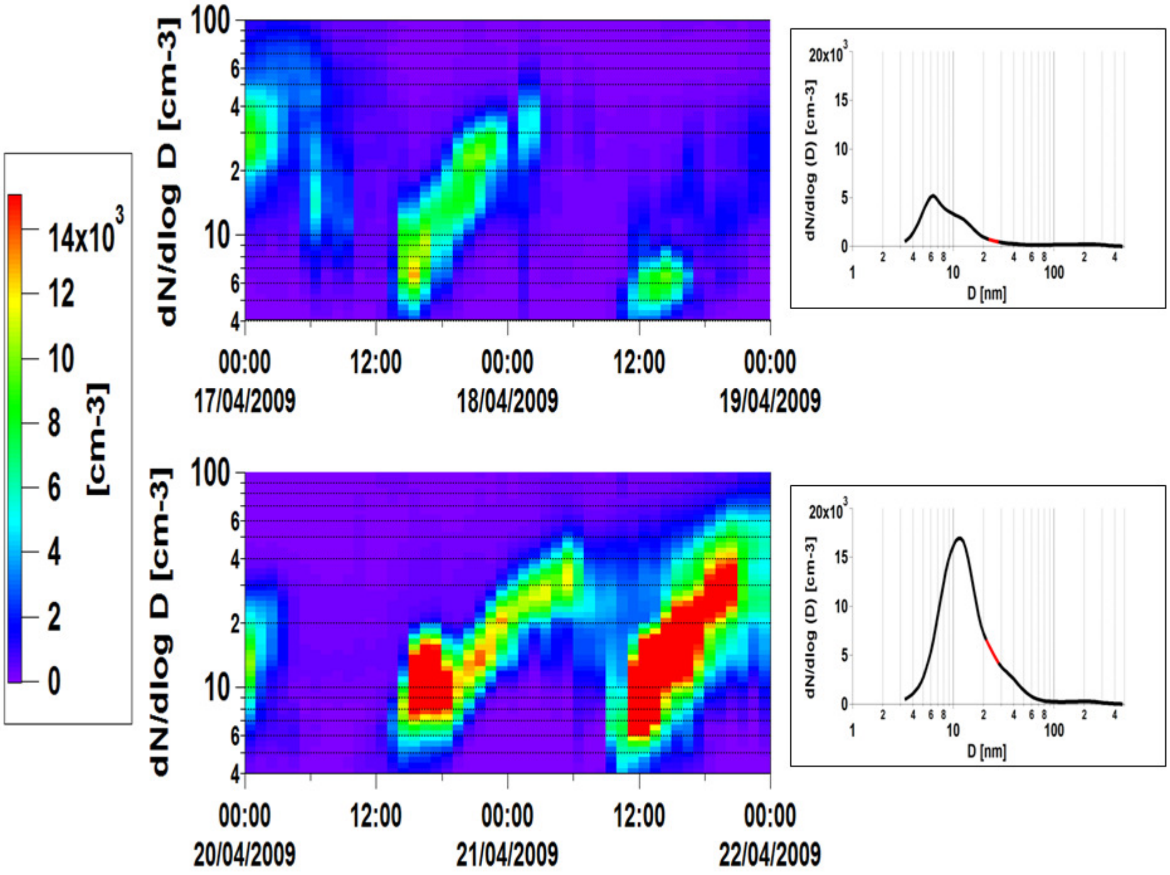

Figure S1f. Results from this study were compared with those reported by Manninen et al. (2010)[31]. It is worth mentioning that the present study covers a broader number of stations (24 vs 10) and a wider measurement period (24 months vs 14). About 55% of the nucleation events classified in the previous study[31] were also detected with our methodology. As an example, four nucleation days classified in Manninen et al. (2010)[31] from the month of April 2008 (SMR station) are reported. Weak nucleation events in particle number concentration and growth (17-18/04/2009, Figure S1g top) are not classified in our study, whereas strong ones (20-21/04/2009, Figure S1g bottom) are. The right hand panels show aerosol size distributions during the beginning of the event (12:00-16:00), and in red the aerosol size bins (17-30nm) used in the K-means clustering used in this study.

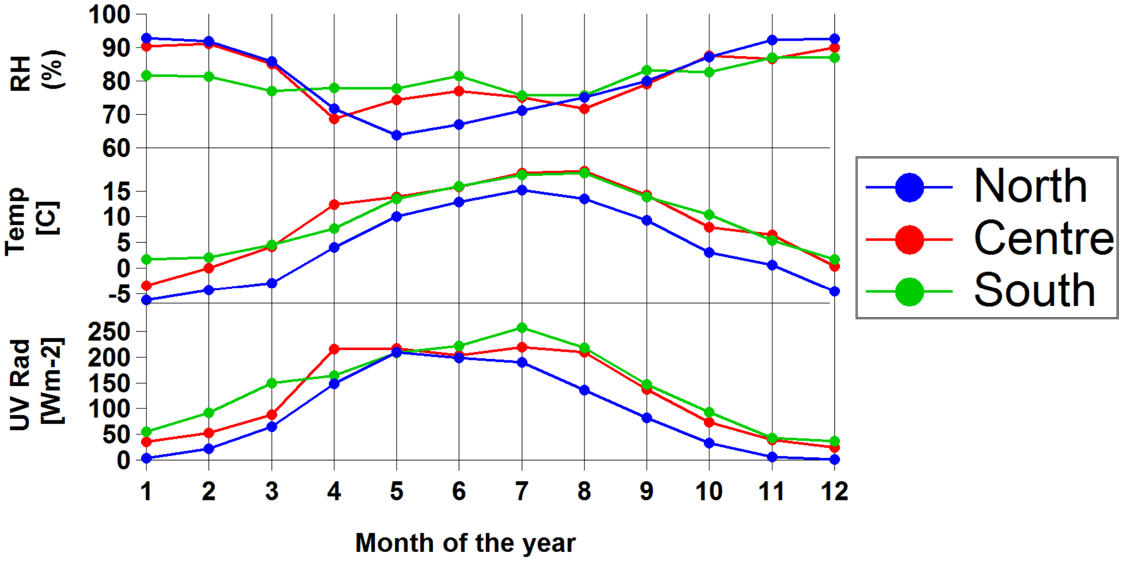

Figure S2a. Average meteorological data across Europe.

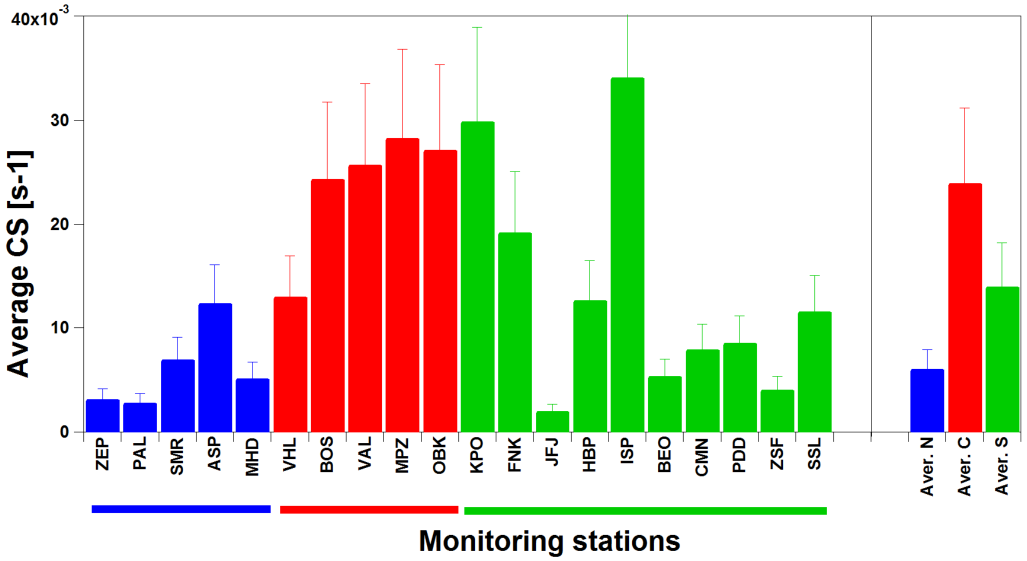

Figure S2b. Average Condensation Sink among the monitoring stations (and averages on the right side, Blue: North; Red: Centre; Green: South).

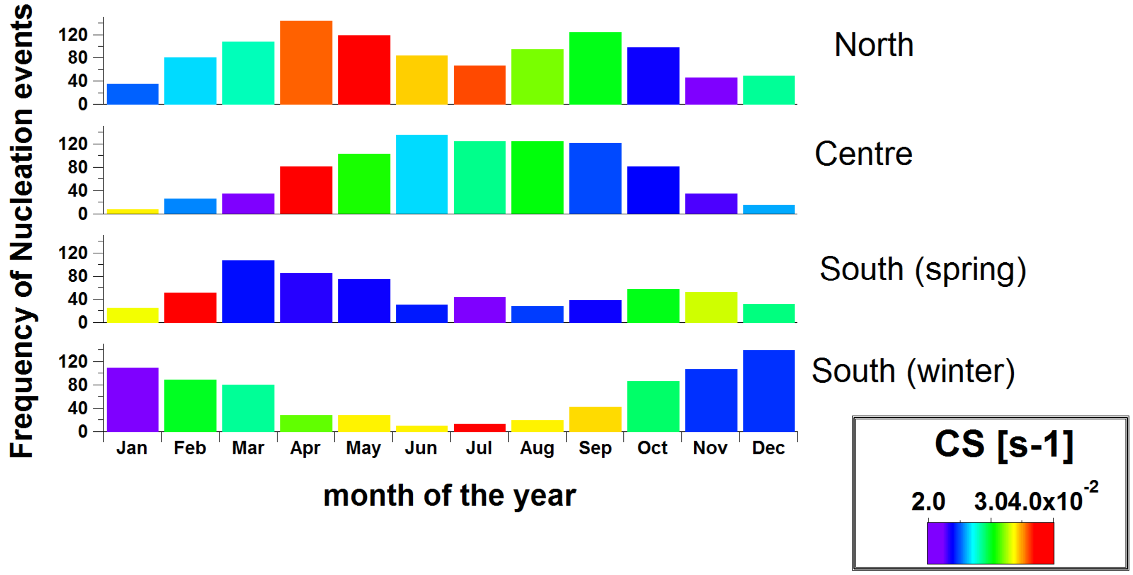

Figure S2c. Annual variation of the CS across the four different European regions.

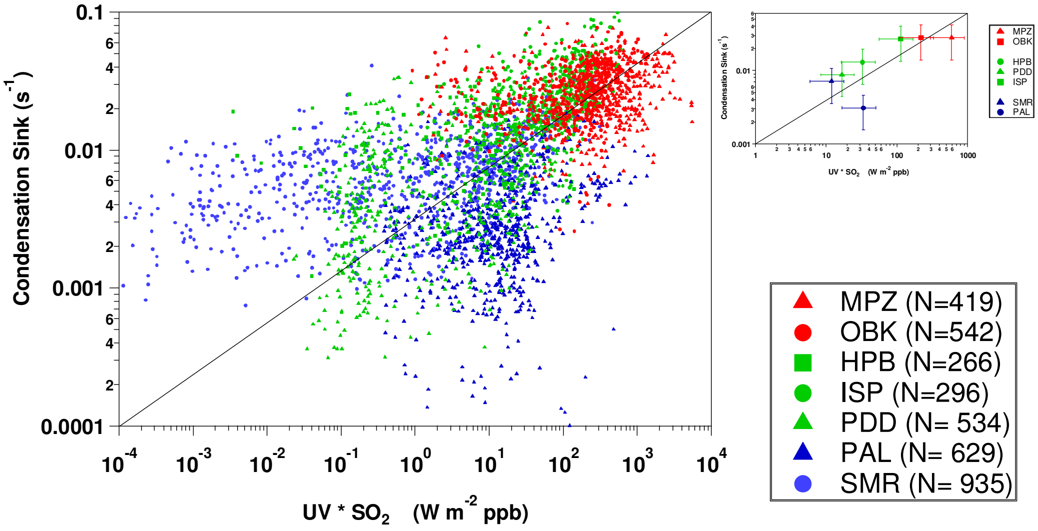

Figure 2d: Relationship of nucleation events at seven European sites with UV * SO_2 (surrogate for H_2SO_4 formation) and condensation sink.

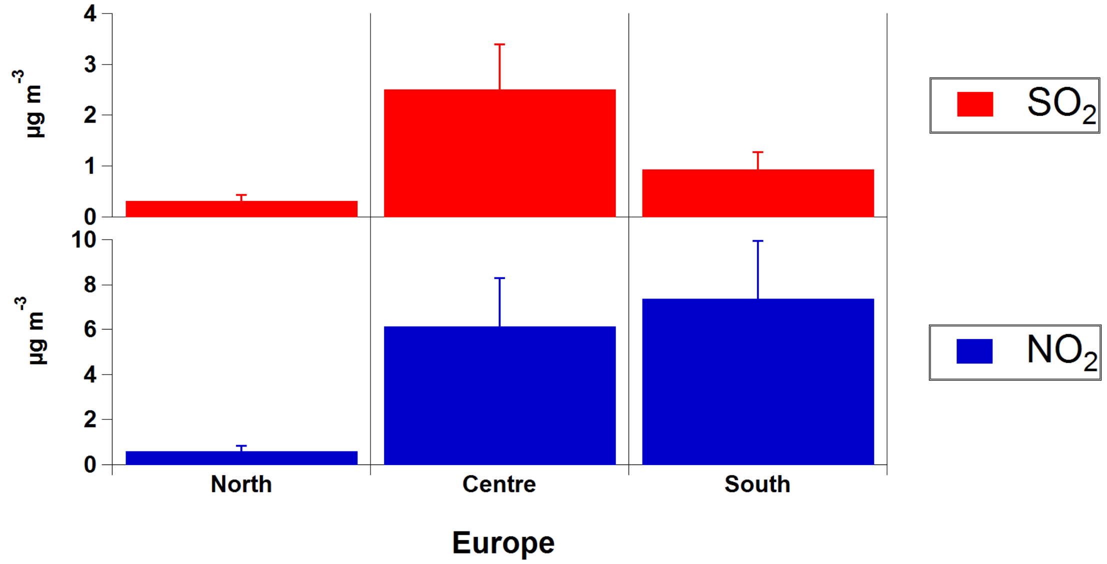

Figure S3a. SO_2 and NO_2 average concentrations for North, Centre and South Europe.

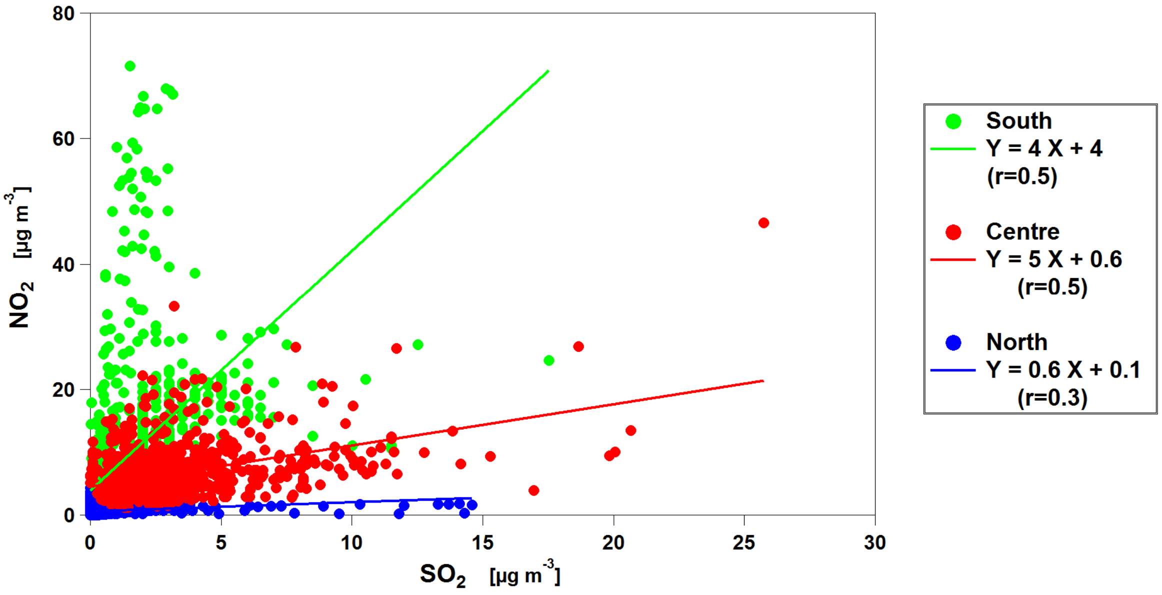

Figure S3b. NO_2-SO_2 correlation plot for North, Centre and South Europe.

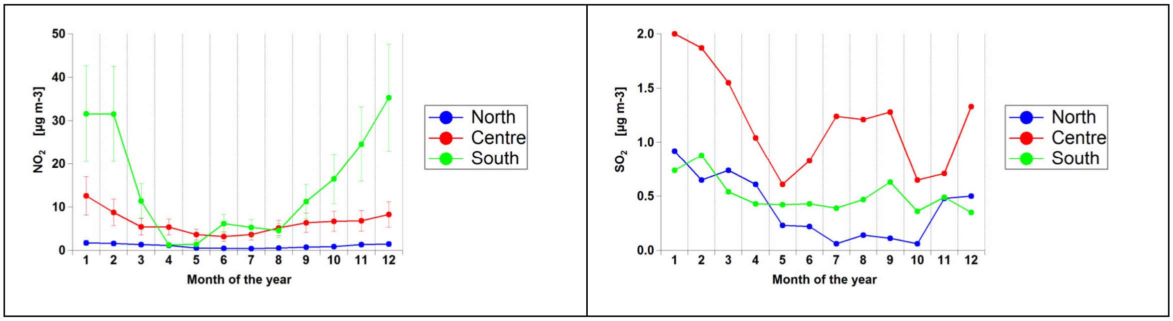

Figure S3c. Annual variation of SO_2 and NO_2 concentrations in North, Centre and South Europe.

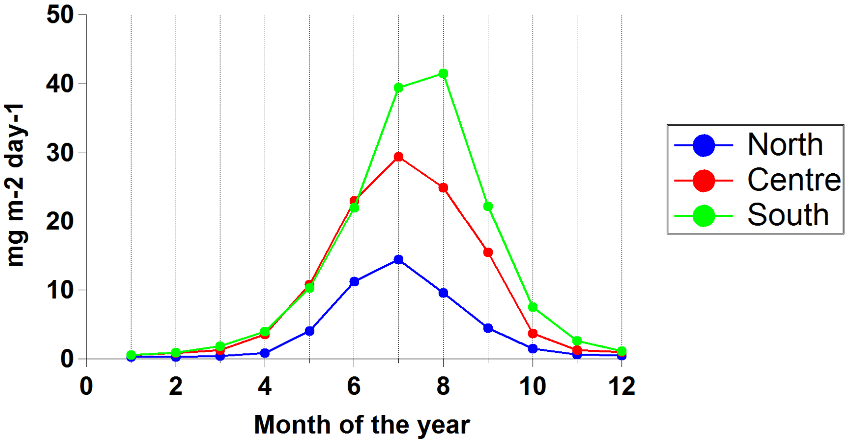

Figure S3d. Annual trend of sum of BVOC fluxes across North, Centre, and South Europe. The annual variation of biogenic VOC (acetaldehyde, acetone, ethanol, formaldehyde, isoprene, methanol, other monoterpenes, α-pinene, β-pinene, propene, sequiterpene) around Europe does not change dramatically across the different stations, with the months of June-July-August having the highest concentrations.

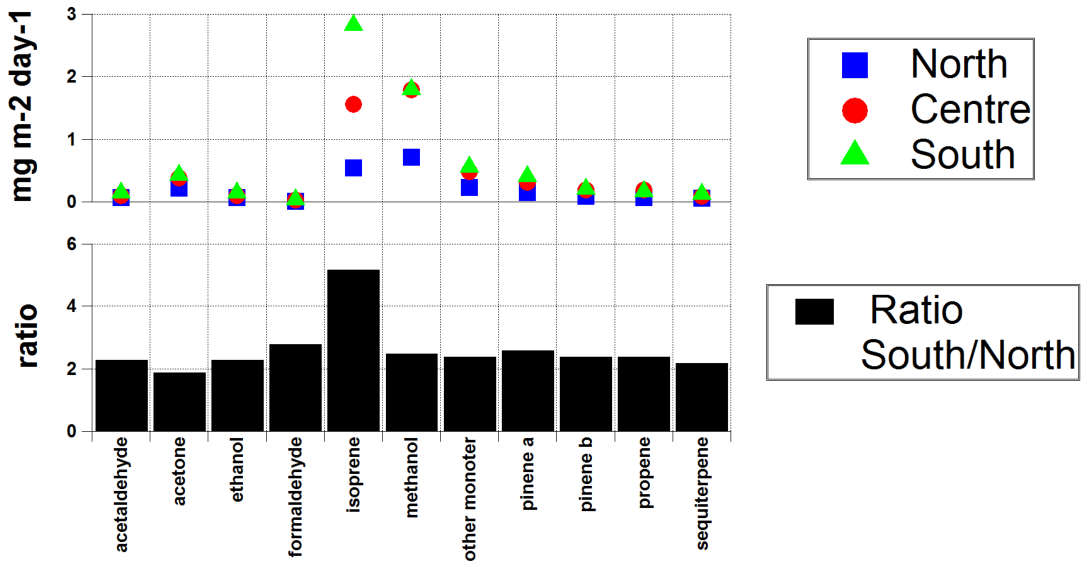

Figure S3e. Average fluxes for BVOC across North, Centre, South Europe, and gradient between South/North Europe. When we take the 24 stations and we plot the average monthly concentrations for the three different regions, we see that a gradient South-Centre-North is often observed, especially for isoprene.

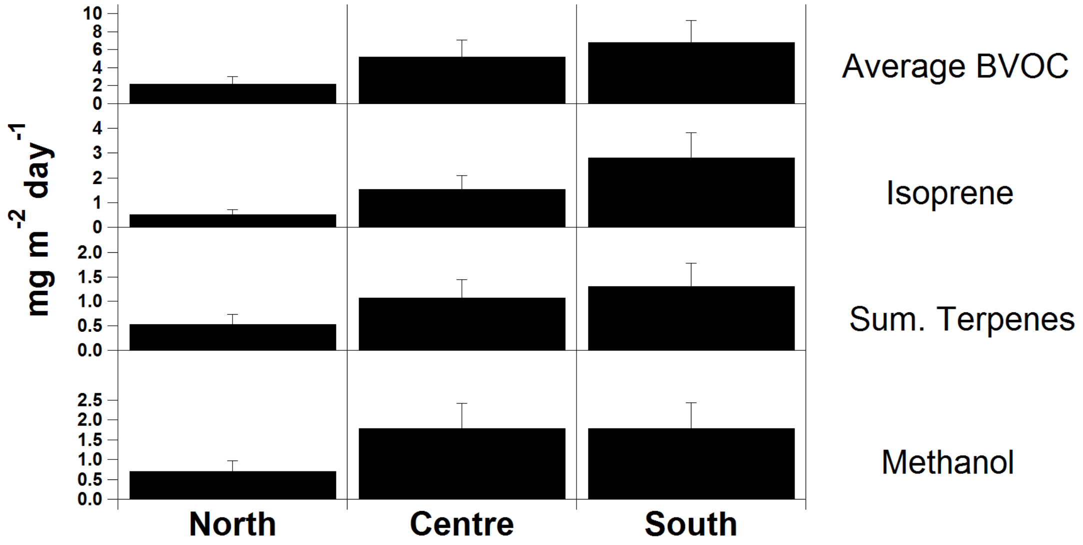

Figure S3f. Average fluxes for selected BVOC across North, Centre, South Europe.

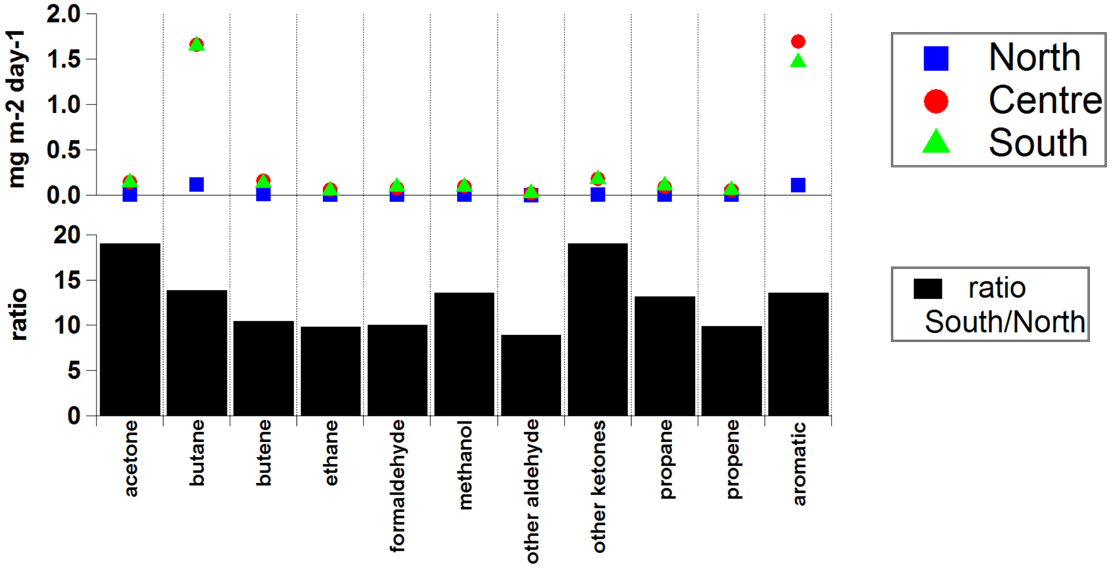

Figure S3g. Average fluxes for VOC across North, Centre, South Europe, and gradient between South/North Europe. The Anthropogenic VOC present very different trends relative to the Biogenic VOC reported before, and describing such variation is beyond the scope of this work. Moreover, the ratio South/North for anthropogenic VOC is higher (about 10-20) than biogenic VOC (about 2), as shown in Figure S4g. Moreover, a clear difference between centre and south Europe can also be seen. Formaldehyde and other aldehydes have a North South gradient, whereas aromatic compounds and butane are higher in the centre part of Europe.

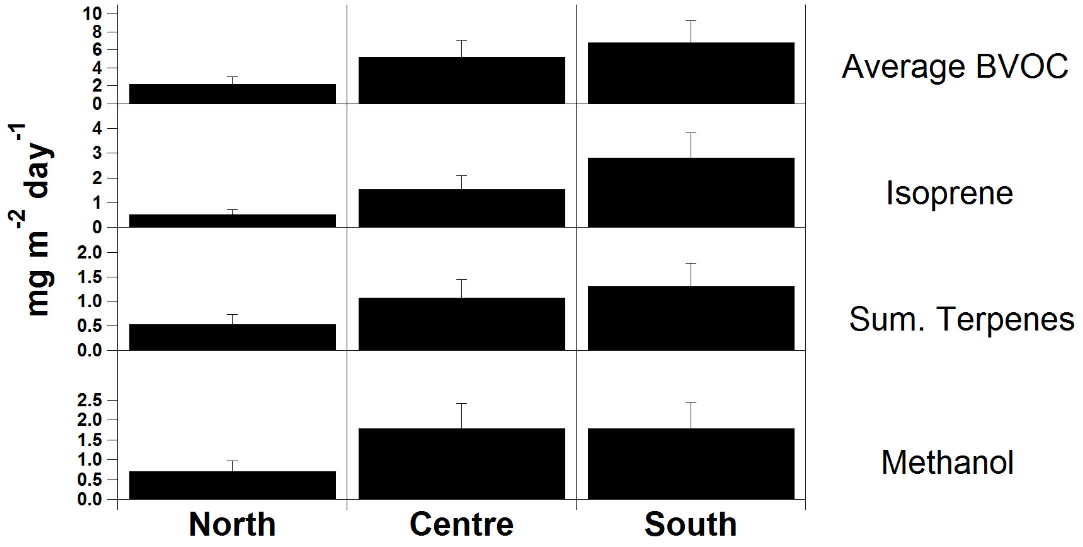

Figure S3h. Average fluxes for selected VOC across North, Centre, and South Europe.

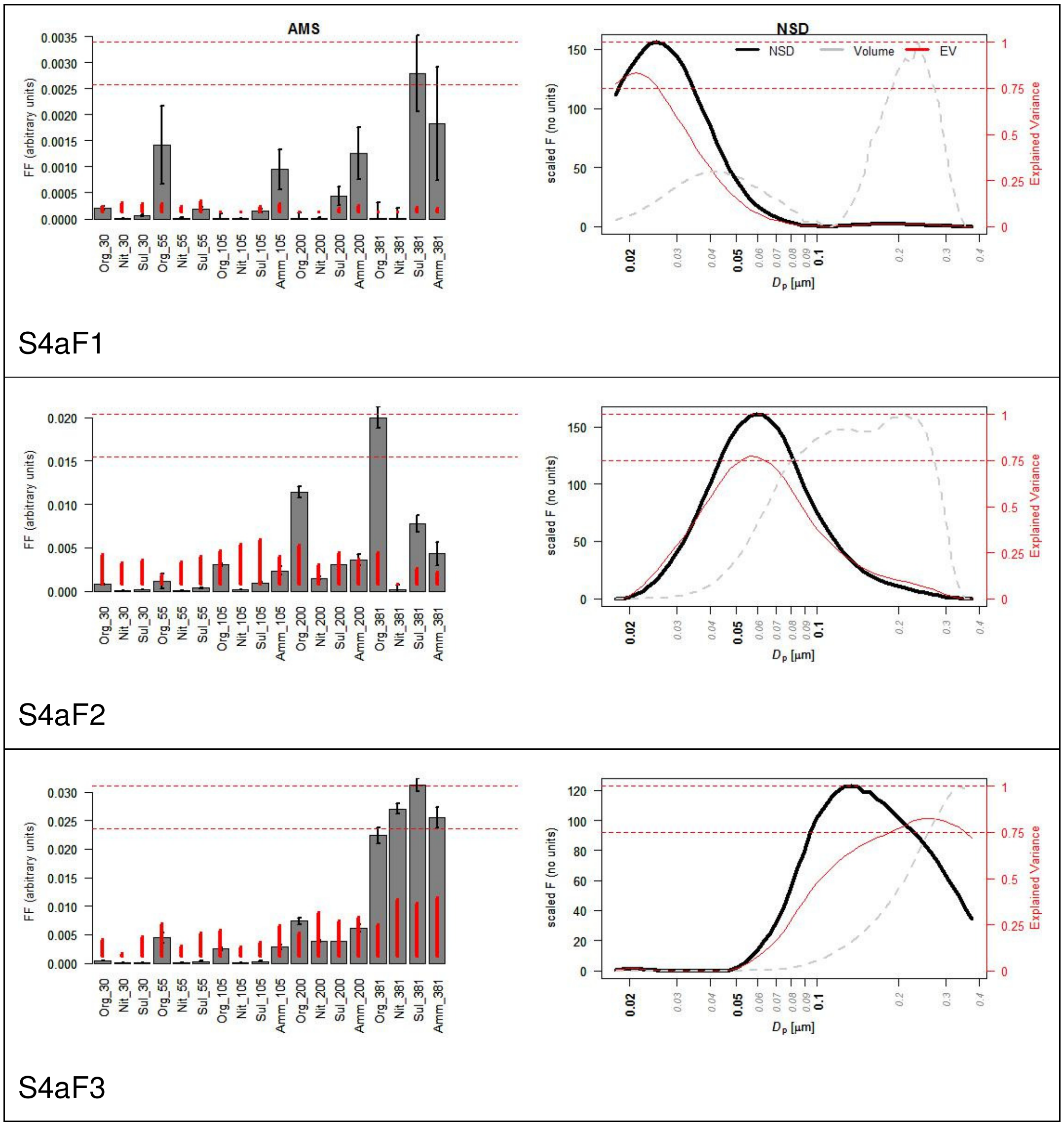

Figure S4a. Three factor PMF solution of combined ToF HR-AMS and SMPS data. The left-hand panels show AMS analytes according to their size bin (30-381 nm). The right-hand panels show the SMPS number and volume size distributions and the explained variation.

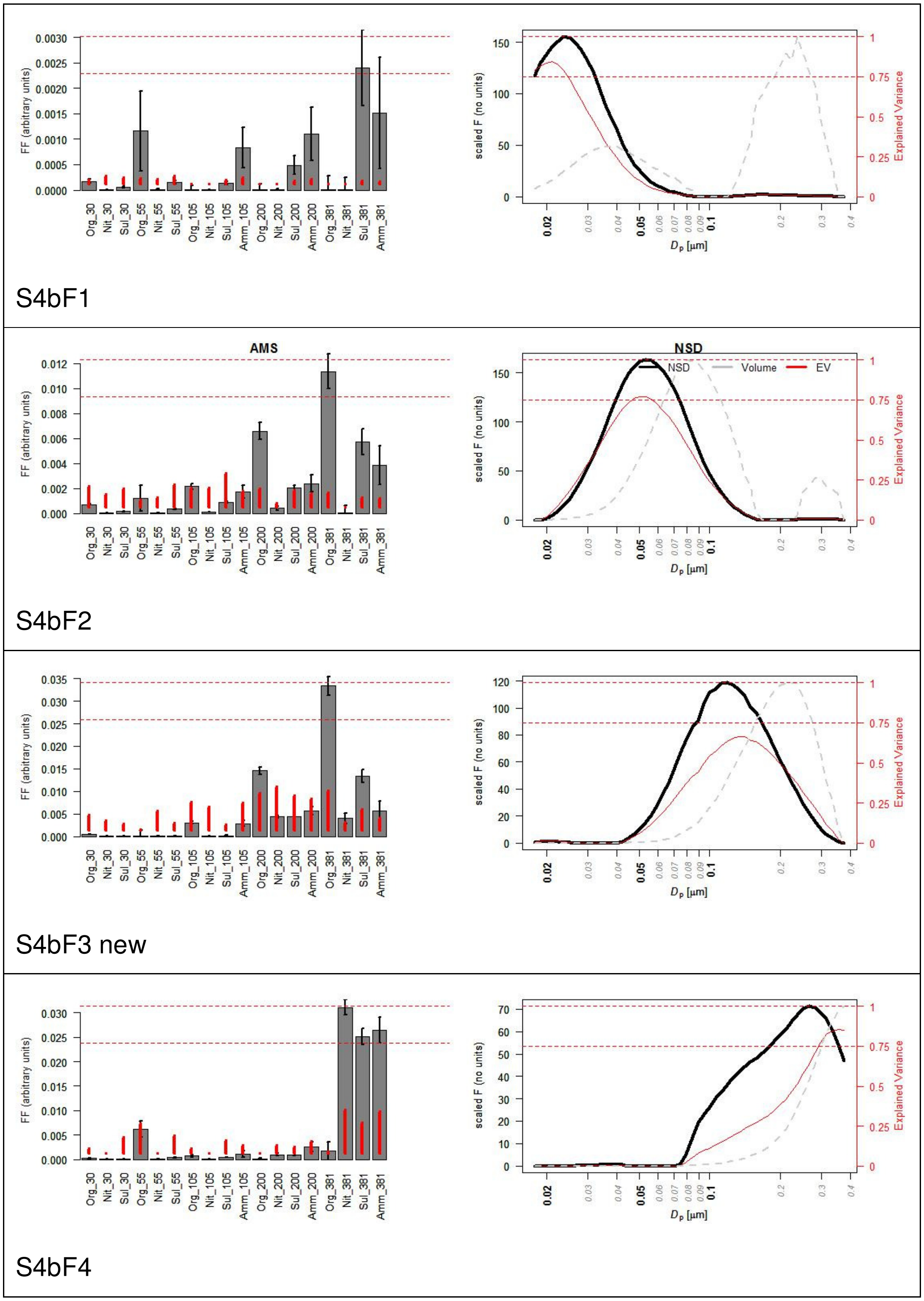

Figure S4b. Four factor PMF solution of combined ToF HR-AMS and SMPS data.

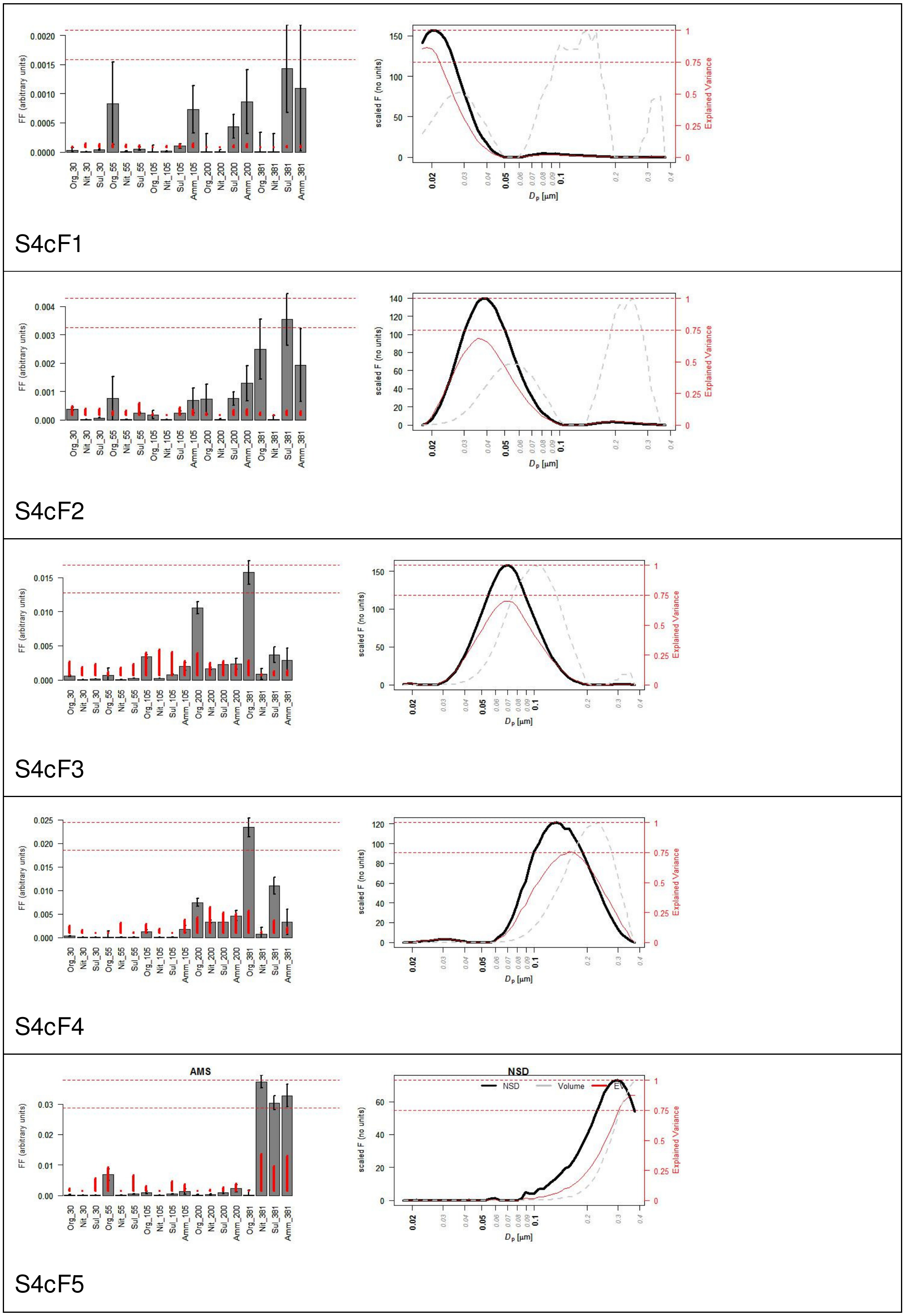

Figure S4c. Five factor PMF solution of combined ToF HR-AMS and SMPS data.

Table S1. Locations and names of stations used in the data analysis. The site altitudes are given in reference to standard sea level. The areas are grouped by European sub-divisions using definitions from Central Intelligence Agency (2009). Country codes are given in the ISO 3166 standard. Right columns are for SMPS, AMS and SO_2 availability at the stations considered.

Station name	Station code	Country	Coordinates, altitude (lat., lon., height.)	SMPS	AMS	Gas SO_2
Nordic and Baltic						
Aspvreten	ASP	SE	58°48'N,17° 23'E, 30 m	V		
Birkenes	BIR	NO	58°23'N, 8°15'E,190 m	V		
Pallas	PAL	FI	67 ° 48'N, 24°7'E, 560 m	V		V
Preila	PLA	LT	55°55',N, 21°0'E, 5 m	V		
SMEAR II	SMR	FI	61 °51'N, 24°17'E, 181 m	V	V	V
Vavihil	VHL	SE	56°1'N, 13°9'E, 172 m	V	V	
Central Europe						
Bösel	BOS	DE	53°7'N, 57°57'E, 16 m	V		
K-Puszta	KPO	HU	46°58'N, 19°19'E, 125 m	V	V	
Melpitz	MPZ	DE	51°32'N, 12°12'E, 87 m	V	V	V
Kosetice	OBK	CZ	49°35'N, 15°15'E, 534 m	V		V
Hohenpeissenberg	HPB	DE	47°48'N, 11°11'E, 988 m	V		V
Waldhof	WAL	DE	52°31'N, 10°46'E, 70 m	V		
Western Europe						
Cabauw	CBW	NL	51°18'N, 4°55'E, 60 m	V		
Harwell	HWL	UK	51°34'N, 1°19'W, 60 m	V		
Mace Head	MHD	IE	53°19'N, 9°53'W, 5 m	V		
Mediterranean						
Finokalia	FKL	GR	35°20'N, 25°40'E, 250 m	V		
JRC-Ispra	ISP	IT	45°49'N, 8°38'E, 209 m	V		V
Arctic						
Zeppelin	ZEP	NO	78°55'N, 11°54'E, 474 m	V		
			High Altitude sites (over 1000 msl)			
Western Europe						
Puy de Dôme	PDD	FR	45°46'N, 2°57'E, 1465 m	V	V	V
Central Europe						
Schauinsland	SCH	DE	47°55'N, 7°55'E, 1210 m	V		
Zugspitze	ZSF	DE	47°25'N, 10°59'E, 2650 m	V		
Jungfraujoch	JFJ	CH	46°32'N, 7°59'E, 3580 m	V		
Balkans						
BEO Moussala	BEO	BG	42°10'N, 23°35'E, 2971 m	V		
Mediterranean						
Monte Cimone	CMN	IT	44°11'N, 10°41'E, 2165 m	V		

Table S2. Initial settings used to investigate the data. For each factor number F, t and v were adjusted until $Q/Q_{theory} \approx 1$; $Q_{NDS}/Q_{NSD\_theory} \approx 1$; $Q_{AMS}/Q_{tAMS\_heory} \approx 1$.

F	**3**	**4**	**5**	**6**	**7**	**8**	**9**
t	0.100	0.100	0.075	0.100	0.075	0.100	0.075
v	0.100	0.075	0.100	0.050	0.075	0.015	0.050
D_{nsd}	0.6	0.475	0.38	0.28	0.235	0.19	0.171
D_{ams}	2.1	2.4	2.75	2.95	3.3	3.8	4.3
Factor Splitting (Yes/No)	N	N	N	N	Y	Y	Y
IM	0.55	0.52	0.51	0.47	0.47	0.42	0.42
IS	1.90	1.92	1.72	1.98	1.84	2.57	2.22
Max(ROT)	0.0038	0.0027	0.0015	0.005	0.004	0.0034	0.0394
